# Application of Acyzol in the Context of Zinc Deficiency and Perspectives

**DOI:** 10.3390/ijms20092104

**Published:** 2019-04-29

**Authors:** Gjumrakch Aliev, Yi Li, Vladimir N. Chubarev, Svetlana A. Lebedeva, Lidiya N. Parshina, Boris A. Trofimov, Susanna S. Sologova, Alfiya Makhmutova, Marco F. Avila-Rodriguez, Sergey G. Klochkov, Pavel A. Galenko-Yaroshevsky, Vadim V. Tarasov

**Affiliations:** 1 Department of Pharmacology and Pharmacy, Sechenov First Moscow State Medical University (Sechenov University), 8-2 Trubetskaya St., Moscow 119991, Russia; tchoubarov@mail.ru (V.N.C.); lebedeva502@yandex.ru (S.A.L.); susanna.sologova@yandex.ru (S.S.S.); tarasov-v-v@mail.ru (V.V.T.); 2 Institute of Physiologically Active Compounds Russian Academy of Sciences, Chernogolovka 142432, Russia; alfiya@ipac.ac.ru (A.M.); klochkov@ipac.ac.ru (S.G.K.); 3 GALLY International Research Institute, 7733 Louis Pasteur Drive #330, San Antonio, TX 78229, USA; 4 Department of Biological and Health Sciences, Texas A&M University-Kingsville, TX 78363, USA; yi.li@tamuk.edu; 5 A.E. Favorsky Irkutsk Institute of Chemistry, Siberian Branch of the Russian Academy of Sciences, 1 Favorsky Str., Irkutsk 664033, Russia; parshina@irioch.irk.ru (L.N.P.); boris_trofimov@irioch.irk.ru (B.A.T.); 6 Universidad del Tolima, Facultad de Ciencias de la Salud, Barrio Santa Helena, Ibagué 730006, Colombia; markos.avila@gmail.com; 7 Department of Pharmacology, Faculty of Pharmacy, Kuban State Medical University, 4 Sedin St., Krasnodar 350063, Russia; galenko.yarochevsky@gmail.com

**Keywords:** Acyzol, carbon monoxide, pharmacological activity, zinc, zinc deficiency conditions

## Abstract

Zinc is one of the most important essential trace elements. It is involved in more than 300 enzyme systems and is an indispensable participant in many biochemical processes. Zinc deficiency causes a number of disorders in the human body, the main ones being the delay of growth and puberty, immune disorders, and cognitive dysfunctions. There are over two billion people in the world suffering from zinc deficiency conditions. Acyzol, a zinc-containing medicine, developed as an antidote against carbon monoxide poisoning, demonstrates a wide range of pharmacological activities: Anti-inflammatory, reparative, detoxifying, immunomodulatory, bacteriostatic, hepatoprotective, adaptogenic, antioxidant, antihypoxic, and cardioprotective. The presence of zinc in the composition of Acyzol suggests the potential of the drug in the treatment and prevention of zinc deficiency conditions, such as Prasad’s disease, immune system pathology, alopecia, allergodermatoses, prostate dysfunction, psoriasis, stomatitis, periodontitis, and delayed mental and physical development in children. Currently, the efficiency of Acyzol in the cases of zinc deficiency is shown in a large number of experimental studies. So, Acyzol can be used as a highly effective drug for pharmacologic therapy of a wide range of diseases and conditions and it opens up new perspectives in the treatment and prevention of zinc deficiency conditions.

## 1. Introduction

Essential trace elements, including zinc as a special one, are the functional basis of many enzyme systems; therefore, their deficiency has a negative effect on cell viability. There are approximately 3000 proteins interacting with zinc [1,2], a considerable portion of those proteins are “zinc finger” transcription factors required for the activation of expression of many thousands of genes [3,4]. Zinc is the only metal present in all classes of enzymes. Zinc is a component of more than 300 enzyme systems involved in the various types of metabolic process, so it is an indispensable participant of many biochemical processes [5,6]. Zinc is essential for the functioning of critical enzymes including DNA and RNA polymerases, respiratory chain, and cytochrome P450, catalase, myelo- and thyroperoxidase, cytochrome oxidase, carbonic anhydrase, carboxypeptidase, as well as the enzyme for the key reaction of heme biosynthesis, etc. [7,8]. Zinc is included in the superoxide dismutase (SOD) structure functions as a powerful antioxidant, which prevents lipid peroxidation (LPO) and protects cell membranes from damage [9]. Zinc induces the biosynthesis of the protective cellular proteins-metallothioneins, thereby it is an antioxidant with reparative effect [10,11]. The function of zinc as an antioxidant and a membrane-stabilizer demonstrates its key role in the prevention of free radical-induced damage in inflammatory processes [12].

Zinc essentially preserves the structure of DNA, RNA, and ribosomes. As a component of the aminoacyl-tRNA synthetases and the protein chain elongation factor, it plays an important role in the translation process and thus, it is indispensable in many key stages of gene expression. Therefore, nutritional or genetic zinc deficiency disrupts the functioning of all cell cycle phases and genetic apparatus function. In fact, zinc deficiency may result in stunted growth and delay in physical and mental development.

Zinc plays a major role in the hormonal status in the human body by directly affecting the production and activity of the hormones of thymus, thyroid glands and pancreas. Zinc regulates several glycolytic processes avoiding diseases, such as type 2 diabetes mellitus and plays an important role in the processes of insulin binding to hepatocytes and lipoprotein synthesis [13,14]. Zinc deficiency arising from reduced absorption in the intestine and excessive excretion by the kidneys contributes to impaired glucose tolerance and can be observed in patients suffering from type 1 and type 2 diabetes mellitus. The interrelation between zinc deficiency and oxidative stress in patients suffering from diabetes suggests effectiveness in combination therapy with antioxidants and zinc in pathology [15,16]. In the case of zinc deficiency, the hypothalamic-pituitary-adrenal system becomes active and synthesis of corticosteroids increases.

Zinc plays a critical role in the human immune system, since it is necessary for the functioning of the thymus, formation of T-cell immunity, neutrophils, macrophages, and natural killer cells [12,17,18,19]. Zinc deficiency activates NF-κB factor and it adversely affects thymulin activity, growth, and functioning of T- and B-lymphocytes, phagocytosis and cytokine production, as well as gene expression of IL-2 and its receptors, resulting in the drastic reduction of organism’s resistance to infection [10,20]. Zinc stimulates the synthesis of anti-bacterial and anti-inflammatory mediators, therefore zinc may be used to prevent and treat inflammatory dermatoses [17]. Zinc participates in the synthesis of retinol-binding protein (RBP), and is required for the metabolism of vitamin A. Zinc has an antiviral effect as it inhibits the replication of nucleic acids of the herpes simplex virus (herpes simplex 1 and 2), encephalomyelitis virus, poliovirus, Coxsackie virus, enterovirus, rhinovirus, and induces apoptosis in the cells transformed by these viruses [21,22,23]. The ability of topical zinc-containing antiseptic creams to prevent the distribution of human immunodeficiency virus (HIV) and Herpes simplex 2 virus has been demonstrated during sexual contacts in macaques [24]. Zinc deficiency reduces resistance to, not only viruses, but also bacteria, fungi, and protozoa, such as *Salmonella enteritidis*, *Lysteria monocytogenes*, *Candida albicans*, *Toxoplasma gondii*, which would increase the risk of incidence of pneumonia, malaria, tuberculosis, measles and other infectious diseases [25,26].

It is noted that therapeutic levels of zinc improve the condition of the patients suffering from sickle cell disease by inducing copper decrease in them. As a result, zinc-containing medications may be promising therapies for Wilson’s disease [12,27]. In neurodegenerative conditions including Parkinson’s disease, it was observed a decrease in the levels of zinc and the dysfunction of prolidase activity [28]. Zinc has antiulcerogenic effects, so it is necessary for the processes of cicatrization, for healing of burns, ulcers of the skin, and mucous membranes [29].

Zinc-containing protein gustin, produced by the parotid gland is responsible for taste sensation, therefore, there are disorders of taste and smell perception under zinc deficiency [30,31,32,33]. Zinc deficiency can be a potential risk factor for dental and periodontal diseases [33]. Zinc is involved in the synthesis of collagen and the formation of bone tissue, calcification processes, cell proliferation, and differentiation. Zinc plays a critical role in the regeneration of skin, growth of hair and nails, secretion of sebaceous glands. It promotes vitamin E absorption and the maintenance of its normal concentration in the blood. Zinc is involved in spermatogenesis and in the functioning of Leydig cells, and maintains normal levels of testosterone, which is a prerequisite for the sexual development of boys [17,34,35,36,37,38,39].

Zinc is involved in hematopoiesis and it promotes the removal of carbon dioxide and the heavy metal salts from the body [40,41]. When included in alcohol dehydrogenase structure, zinc has catalytic and structural functions and plays an important role in alcohol metabolism, so that zinc deficiency may increase susceptibility to alcoholism, particularly among children and adolescents.

Deficiency of this element is associated with the development of metabolic disorders, which are accompanied by multiple severe abnormalities (coronary heart disease (CHD), atherosclerosis, hepatitis, pulmonary disease, skin, gastrointestinal disorders, and the destructive changes of periodontal tissues, etc.) [42,43,44,45,46,47]. However, a delay in growth and puberty, immune disorders, and cognitive disorders are the main clinical manifestations of zinc deficiency in the human organism. 

## 2. Zinc Deficiency Conditions (ZDC)

The first discovery of the biological role of zinc appeared in the XIX century, when Raulin first showed the key role of zinc for the growth of *Aspergillus niger* in 1869 [48]. In 1934, the signs of zinc deficiency were discovered in laboratory rats, including growth retardation, hair loss, thickening and hyperkeratinization of the epidermis and testicular atrophy [49]. Human ZDC was widespread throughout the world and was first described by A. Prasad in the 60‘s of the last century [50]. Today this problem is a subject of numerous studies worldwide. According to the reports, more than 2 billion people currently suffer from zinc deficiency [12,16,51,52]. In developing countries, ZDC is a critical issue among the poor and elderly [16,53,54,55] because of a diet low in zinc [16]. Zinc deficiency is one of the key factors of morbidity in developing countries with high mortality rates [56].

The causes of zinc deficiency are mainly exogenous, such as alimentary (diseases of gastrointestinal tract, liver and kidneys, pregnancy, physical exercises, stress, inflammation, and injuries), iatrogenic (imbalanced or prolonged parenteral nutrition, prolonged treatment with L-histidine, D-penicillamine, cytostatics, estrogens, corticosteroids, diuretics) or industry-related (excess of heavy metals: lead, cadmium, chromium in the environment) [51,53,57,58,59,60]. Furthermore, it is possible to distinguish the Primary Zinc Deficiency associated with insufficient intake of zinc from foods and Secondary Zinc Deficiency, resulting from diseases that reduce intestinal zinc absorption or increase its excretion. They are such diseases as celiac disease, cystic fibrosis, and other gastrointestinal malabsorption syndromes [61], chronic inflammatory diseases of the bowels [62], kidneys and liver, hemolytic anemia, such as thalassemia and sickle cell disease [63,64,65], and also intestinal parasites, alcoholism, stress, and others. ZDC may occur in the antenatal and postnatal periods of ontogenesis.

The effects of zinc deficiency on fetal development during pregnancy were described in the 20th century [66,67,68]. About 15–20% of pregnant women with zinc deficiency result in newborn defects, such as hydrocephaly, microphthalmia, anophthalmia, cleft palate, curvature of the spine, formation of hernia, heart defects, delay of psychomotor, mental and speech development, persistent behavioral changes, disorders of the immune system, etc. Zinc deficiency is observed in premature infants and children with low birth weight. The literature suggests a direct correlation between zinc deficiency and pathology of symptoms in pregnancy and delivery, such as toxicosis, uterine bleeding, premature birth, miscarriage, etc. The development of ZDC is the result of the use of oral contraceptives. Alimentary deficiency of zinc in children shows itself as iron-deficient anemia, hepatosplenomegaly, dwarfism, and hypogonadism (Prasad’s disease). Common manifestations of severe zinc deficiency include diarrhea. The evidence shows that the administration of zinc-containing supplements results in a clinically significant reduction of the duration and severity of diarrhea. Zinc improves the absorption of water and electrolytes in the GI tract, regeneration of the intestinal epithelium and restoration of its function, increase of the level of enterocyte brush border enzymes, activation of immunologic processes including cell-mediated immunity and antibody secretion [12,69,70,71]. ZDC in adolescents are accompanied by behavioral disorders, disorders of taste and smell, which promote the development of persistent addiction to toxic substances. Zinc deficiency can be associated with depression, emotional liability, impaired ability to concentrate, memory loss, development of peripheral neuropathies, etc. [72,73].

Zinc deficiency is observed in the patients suffering from atherosclerosis and rheumatism. It plays an important role in the pathogenesis, progress, and outcome of inflammatory diseases of the uterine appendages. Zinc metabolism disorders accompany gastrointestinal diseases (chronic gastroduodenitis, gastroduodenal ulcer, etc.) and skin diseases (acne, seborrhea, alopecia, psoriasis, etc.). Zinc levels are reduced in the blood of the men suffering from benign prostatic hyperplasia [74,75]. Natural zinc deficiency is one of the major environmental factors that adversely affect the development and progress of tuberculosis [76,77].

Animal studies have shown that glucose uptake by cells in eye lens is impaired in the conditions of zinc deficiency, which is one of the possible causes of cataracts. In the case of age-related macular degeneration, it has been demonstrated that zinc reduces the risk of vision loss [12]. Currently, a relationship between zinc deficiency and the increased occurrence of cataracts among people with diabetes is demonstrated [78]. ZDC accompanies chronic alcoholism that leads to a significant reduction in liver detoxification capacity, due to the deactivation of zinc-dependent alcohol dehydrogenase. ZDC increases lesions of the nervous, urinary, cardiovascular, and other vital body systems. The role of zinc deficiency in chronic alcoholism and in the development of alcoholic psychosis is significant [78]. The efficacy of zinc sulfate for the therapy of endotoxicosis and alcoholic steatohepatitis has been demonstrated in rats [79]. It is shown that zinc deficiency may be associated with psycho-emotional and physical stress, which affects people of dangerous trades, including the personnel that belong to the military forces [80].

## 3. Diagnostics of Zinc Deficiency

For the purpose of ZDC diagnostics, the determination of zinc in plasma, erythrocytes, urine or hair is used. Other approaches are based on measuring serum concentration of zinc-dependent enzymes, carbonic anhydrase, SOD, lactate dehydrogenase, alkaline phosphatase, metallothionein, and retinol-binding protein [30,31]. However, zinc content in granulocytes and lymphocytes more accurately reflects the state of zinc metabolism [81]. Quantitative analysis of the alkaline phosphatase activity of granulocytes, plasma thymulin, and IL-2 expression in mononuclear cells are also effective diagnostic tests to assess zinc deficiency [12]. In order to register zinc content, the following methods are used: flame atomic absorption spectrometry (FAAS), atomic absorption spectrometry using a graphite furnace (GFAAS), mass spectrometry and atomic emission spectrometry with inductively coupled plasma (ICPMS, ICP-AES), neutron activation analysis (NAA), proton-induced X-ray emission (PIXE), and anodic stripping voltammetry (ASV) [82,83,84,85]. The study of biomarkers of zinc and improving the diagnostics of Zn deficiency in the body are actual problems.

## 4. Acyzol in the Treatment and Prevention of ZDC

Application of food supplements can significantly increase zinc intake to the organism. However, the issues of inadequate zinc intake with food are insufficiently studied [16]. Zinc-containing drugs have the advantage of the absence of toxic and teratogenic effects, and are therefore useful in the treatment and prevention of ZDC [12]. Treatments by zinc-containing drugs improve neuropsychiatric functions, which increases a person’s capacity for work, perception of complex shapes, visual memory, attention, concept formation, and the selection of key phrases [10]. Zinc-containing drugs improve the general condition of the patients suffering from coronary artery disease; in these patients, reduction in heart rate, decrease of dyspnoea, pain syndrome, the content of total lipids and cholesterol are observed. On the background of the intake of zinc-containing drugs, the duration and severity of cold symptoms are reduced; that may be associated with the decrease in the level of anti-inflammatory cytokines antioxidant and anti- inflammatory properties of zinc [86]. Zinc preparations are used in oncology practice because of their immunomodulatory effects. Zinc-containing drugs used in clinical practice are unfortunately not always sufficiently effective for the treatment and prevention of ZDC, which is likely associated with their low bioavailability.

**Figure 1:**
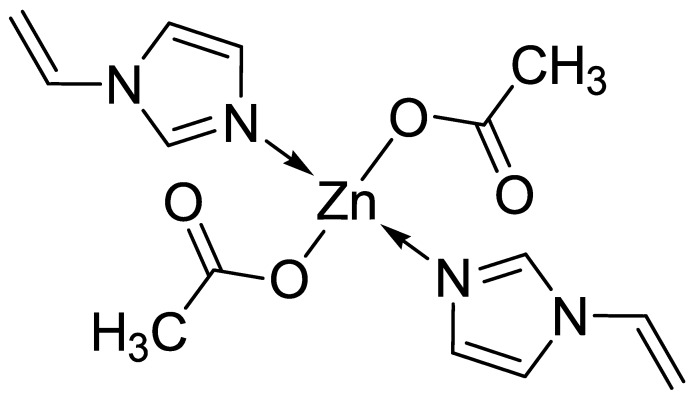
Structural formula of Acyzol.

Acyzol [bis(1-vinylimidazole)zinc diacetate] is a zinc-containing drug with high bioavailability, low toxicity and good tolerability, which was synthesized in the Irkutsk Institute of Chemistry of Siberian Branch of the Russian Academy of Sciences. The structural formula of the compound is shown in Figure 1. Results of Acyzol study, testifying a wide range of its pharmacological effects, allow to suggest that the interaction of zinc with the best possible for him complexing agent-vinylimidazole (azole group ligand) provides bioavailability of Acyzol [87] and underlies its effectiveness. For clinical use, the drug is produced in an encapsulated form of 120 mg and in the form of solution for intramuscular injection in 1 mL ampoules of 60 mg/mL by CJSC “Makiz”. Acyzol maximum concentration in the blood is reached in 20−30 min after the intramuscular injection and in 1 h after oral administration of 1 mL (60 mg) of the drug and remains in the blood plasma for 4.5 h. The half-life period is 1−1.5 h. The drug is mainly metabolized by the liver. In rare cases, Acyzol can cause metallic taste in the mouth, nausea and headache, passing after discontinuation of the drug.

Acyzol has a large therapeutic window and it is safe under prolonged (90 days) daily intramuscular injection, intragastrically and peroral administration to experimental animals including mice, rats, and dogs. Acyzol application does not cause pathological changes of biochemical parameters and has no negative effect on the physiological systems of the body and on the structure of the internal organs. The tested formulations are non-irritating; they do not cause allergic reactions, do not have embryotoxic and teratogenic properties, do not affect reproductive function in animals. Acyzol is protected by 14 patents of the Russian Federation.

Acyzol is a powerful antidote against acute poisoning with lethal doses of CO, carbon monoxide or carbon oxide, and other products of combustion. It accelerates their elimination from the body and reduces the severity of the intoxication, which contributes to the success of subsequent medical interventions (oxygenobarotherapy, symptomatic drug therapy, etc.) [88]. The drug is recommended for use in the conditions of underwater, aerospace and mining works, as well as in the areas of fires and in the cases of ailments caused by car exhaust and smog. It is an effective protective agent that allows providing the required level of organism resistance to the toxic effects of carbon monoxide over a wide range of concentrations and exposure times of the poison, which is the most important for liquidators of the consequences of accidents accompanied by fires [89,90]. Clinical studies have shown high efficacy of antidotal properties of Acyzol. This is proved by the decrease in CO initial concentration by 2 times in a patient’s blood an hour after injection and by the increase of the half-life of carbon monoxide by 5.3 times compared with conventional therapy [91]. Under a threat of poisoning by carbon monoxide and other products of thermal oxidative degradation, Acyzol is used as a prophylactic remedy; while in the cases of poisoning of various severities caused by these substances, Acyzol is used as a therapeutic agent.

Effectiveness of Acyzol for the prevention and treatment of toxicohypoxic encephalopathies (TSEs), aggravating the progress of acute intoxications caused by CO and other combustion products, has been studied in experiments and in clinical trials. Administration of Acyzol (intramuscular injections of 6% solution 3 times daily for 7−10 days) to patients suffering from CO poisoning is beneficial for reduction of neurological complications. The favorable outcome from TSEs is due to an increase in the CO elimination, correction of non-specific changes in the blood cell composition, stabilization of peroxidation of lipids (POL) and antioxidant system (AOS) POL/AOS, and the elimination of compensatory reactions of the blood circulatory system [91,92,93].

The mechanism of Acyzol action is due to its influence on the cooperative interaction of the hemoglobin subunits, thereby resulting in the reduction of the relative hemoglobin affinity to carbon monoxide and improvement of the oxygen-binding (decreasing of Hill’s constant) and gas-transport functions of the blood [90,94]. The experimental works conducted in the Institute of Toxicology of the Federal Medical and Biological Agency (Saint-Petersburg, Russia) demonstrated that Acyzol modifies interactions of protein complexes in the hemoglobin molecule in a non-electrolytic way, which facilitates the oxygen addition reaction and accelerates decomposition of carboxyhemoglobin [92].

**Figure 2:**
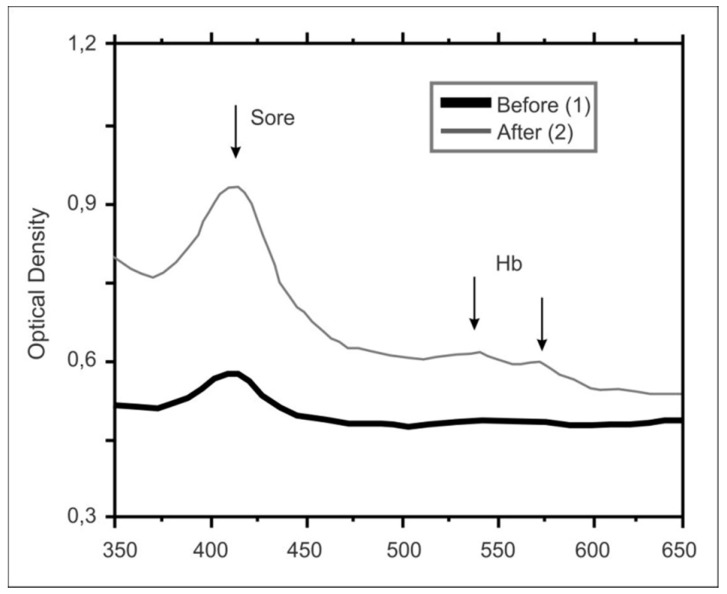
Absorption spectra of blood smears before and after addition of Acyzol (registration using the method of bio-crystallization of Acyzol (1:1500 and 1:3000) with the blood) [with permission from 95]. Annotation: 1) absorption spectra of a blood smear of a healthy donor. 2) absorption spectra of a blood smear after supplementation of Acyzol.

A direct chemical effect of the main component of the drug on the blood has been proved by the bio-crystallization method. The effect shows itself in the conversion of heme and globin part of the blood hemoglobin into the product, having an altered protein structure. In this case, the oxyglobin characteristic spectrum is observed. It is consists of the following absorption bands: 414 nm (Soret band), and two ones in the areas of 540 nm and 575 nm (α and β-bands). These α- and β-bands almost completely disappear from the spectrum after interaction of Acyzol with blood, indicating the changes of the heme part of hemoglobin, due to the influence of Acyzol (Figure 2) [95]. In addition to optimization of the mode of oxygen intake, Acyzol as a zinc-containing drug provides a balance of redox processes in cells, acting as a universal regulator of energy metabolism. It has membrane-protective properties, prevents the formation of highly reactive forms of oxygen by heavy metals, which enter the organism as part of exhaust gases or air pollution [91,96].

Acyzol is a highly effective zinc-containing drug that provides anti-inflammatory, reparative, detoxifying, immunomodulatory, and bacteriostatic effects. Experimental and clinical studies demonstrate a large therapeutic window of Acyzol. In addition, Levedeva et al. showed the hepatoprotective, adaptogenic, antioxidant, and other cardioprotective activity of Acyzol [97]. The drug maintains the physiological characteristics of the respiratory system, as well as the functioning of the human cardiovascular and urinary systems [40]. 

Interestingly, animal models of hypoxia showed a protective effect of Acyzol (at 38−176% compared to animals of the control groups). Briefly, Acyzol was administered in a wide dose range (10–150 mg/kg) in mice under various acute hypoxic conditions including hypobaric hypoxia (AHBH), hypoxia with hypercapnia (AHwHc), and hemic hypoxia (AHeH) (see Table 1) [98,99]. 

Acyzol, as a highly effective antihypoxic drug, protects the body in the conditions of low oxygen partial pressure and insufficient oxygenation of hemoglobin, and provides resistance to hypoxia of organs (brain, myocardium, liver), which are the most sensitive to it [91,94].

Experimental and clinical studies demonstrate the possibility of creating Acyzol formulations for external use (ointments, creams, pastes, gels, solutions), which will be effective in the treatment of skin diseases (dermatitis, dermatosis, trophic ulcers, etc.) and diseases of mucous membranes (stomatitis, periodontitis, balanoposthitis, etc.).

The presence of zinc shows that Acyzol may be used as a promissory for the treatment and prevention of ZDC including Prasad’s disease, immunodeficiencies, alopecia, allergodermatoses, prostate dysfunction, psoriasis, stomatitis, periodontitis, delayed mental and physical development in children, etc. At present, the effectiveness of Acyzol for ZDC correction has been shown on the huge factual material [42]. 

The use of Acyzol formulations as a pharmacologic agent for pneumonia treatment resulted from acute poisoning with neurotropic agents significantly reduced the mortality rates (see Table 2) [91,99].

**Figure 3:**
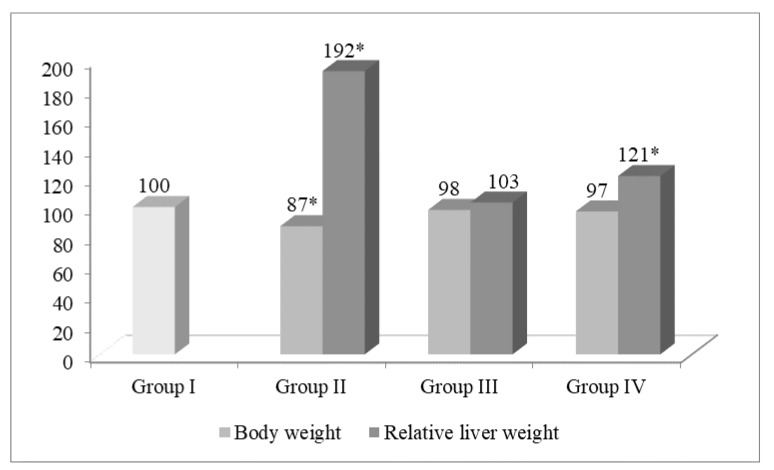
Body weight and relative liver weight in CCl_4_-intoxicated rats and in animals treated with Acyzol and Silymarin. Notes: *—significant differences compared to intact animals at *p* < 0.05. Data are presented in % relative to the intact group, taken as 100%. Relative liver weight is expressed as liver weight, mg/100 g of body weight.

**Figure 4:**
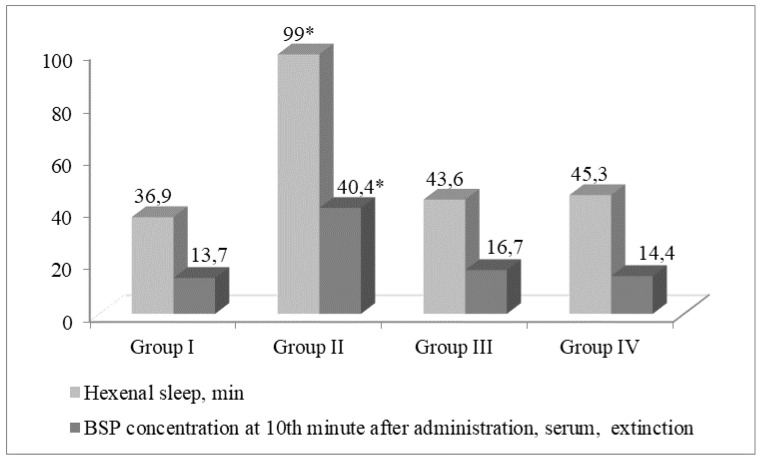
Liver function in CCl4-intoxicated rats and in animals treated with Acyzol and Silymarin. Notice. *—significant differences compared to intact animals at *p* < 0.05. group I—intact. group II received equivalent amount of distilled water. group III received Acyzol at a dose of 10 mg/kg diluted in water. group IV—received Silymarin at a dose of 100 mg/kg.

Similar hepatoprotrotective effect of Acyzol and Silymarin was shown in the preclinical studies, thus providing a rationale for use of Acyzol in complex therapy of toxic hepatitis and hepatosis (Figure 3 and Figure 4) [100].

The study of cardioprotective effectiveness of Acyzol demonstrated pronounced therapeutic effect of the drug in the case of experimental catecholamine myocarditis. Increased contractile activity of the myocardium and decrease in the overload of the right heart were observed; that led to the regression of inflammatory changes in the cardiomycytes, reduced severity of cytolysis and increased intensity of tissue respiration [97]. In the context of the development of the experimental infarction caused by ligation of the left coronary artery in white rats, Acyzol in a dose of 60 mg/kg showed anti-ischemic effect, increasing myocardial contractile activity and accelerating atrioventricular and intraventricular conduction (Table 3) [101].

During therapy with Acyzol, an increase in the amplitude of the R, T waves was observed compared to the normal state, which may indicate an increase in the contractile activity of the myocardium. A decrease in the amplitude of the P, R, T waves and an increase in atrioventricular conductivity were also noted (significant shortening of PQ interval in the observation period of 1 h compared with the control) [101]. 

The results of the study of hemodynamics and organ-specific characteristics on the model of cardiopathic amyloidosis induced by administration of Freund’s adjuvant to male Wistar rats weighing 350−400 g and aged 18−24 months show that administration of Acyzol 30 mg/kg intragastrically for 60 days causes a positive preventive effect [102].

The study of psoriatic patients carried out by Abramova TV, Batkaeva EA (2006) showed the efficacy of Acyzol when it administered in a dose of 120 mg 1 time a day for 10 days on the background of the conventional therapy for that disease that included hyposensitization drugs, vitamins, hepatoprotective agents, sedatives, ultraviolet irradiation, and external ointment treatment [103]. Administration of Acyzol (into the complex therapy of the patients suffering from psoriasis) increased the frequency of pronounced therapeutic effect up to 80% along with the decrease in PASI scores by more than 70% in 77.9% of patients on the background of good tolerability. Acyzol showed an absence of side effects and reduction of the terms of treatment, compared with the control group of patients treated with conventional therapy [103].

The course of Acyzol application for 2 months in a dose of 2 capsule (40 mg Zn) per day by military men with a low level of physical development, weight deficit, and increased morbidity level has improved functional status and increased the adaptive capacity of the body. In particular, pulmonary capacity, chest circumference at inspiration and expiration, Erismann index and other functional and physical characteristics of the body have increased [104]. The course of Acyzol application by military men restored the normal level not only of zinc but also of other essential chemical elements (Ca, P, K, Na, Mg, Fe, Si, Se, V, I) in the background of reduction of conditionally essential and toxic elements [104]. That proves the unique ability of zinc to provide a universal sanogenetic effect, which shows itself in the restoration of elemental homeostasis in cases of zinc deficiency and imbalance of the elemental status [96,104,105,106].

Experimental and clinical studies during the treatment of periodontal disease (gingivitis, periodontitis, and periodontal disease) of varying severity have shown that the use of Acyzol is accompanied by a rapid recovery and stable remission, both in an experimental laboratory and in the clinic [107]. The high efficiency of a toothpaste and mouthwash, which are based on Acyzol and used in the complex treatment of inflammatory periodontal diseases, has been showed [29,108]. These observations were accompanied by positive dynamics of dental indices (Table 4) [108].

When patients suffering from type 2 diabetes received Acyzol, a decrease in their blood glucose levels was observed, a month after the start of the treatment [108].

Prophylactic administration of Acyzol is an effective way of the toxic effect correction in the case of chronic lead poisoning. It has been shown that, subcutaneous and intragastric administration of Acyzol in a dose of 30 mg/kg to male Wistar rats reduces lipid peroxidation (LPO) processes, increases the activity of the antioxidant defense enzymes (catalase) in blood plasma and reduces malondialdehyde (MDA) content in the membranes of erythrocytes (Table 5) [40].

Acyzol as an antihypoxic agent can be used for the treatment of many diseases involving ischemia and hypoxia of viscera, i.e., in medical and surgical practice, in urology, gynecology, dermatology, and for the treatment of functional impotence. Due to its antihypoxic and antioxidant effects, Acyzol can be used in emergency medicine, in surgery for the correction of metabolic acidosis, and for the protection of cell membranes from the effects of LPO products. Acyzol can be recommended for introduction in the obstetric practice as an effective remedy for fetal hypoxia.

The application of Acyzol is promising for sports medicine, because the drug increases subjects’ performance and endurance in the training process, owing to zinc replenishment in the enzyme systems, optimization of tissue respiration, and the improvement of the oxygen binding properties of the blood. Acyzol can be recommended for regenerative medicine as a drug that enhances the level of functional reserves of the organism [104].

## 5. Conclusions

Zinc-containing preparation of Acyzol acts as a highly effective drug for the pharmacologic therapy of a wide range of diseases and conditions, including hypoxia, toxicohypoxic encephalopathy, periodontal, immune, and heart pathologies. The use of Acyzol opens up new perspectives in the treatment and prevention of ZDC. 

## Figures and Tables

**Table 1:** Effect of administration (i/p) of Acyzol on the lifespan of mice during acute hypoxia.

Hypoxic Model	Dose, mg/kg, i/p					
	5	10	25	50	100	150
AHBH	95 ± 6	216 ± 14 *	276 ± 18 *	222 ± 9 *	162 ± 12 *	111 ± 8
AHwHc	-	103 ± 4	138 ± 7 *	199 ± 4 *	174 ± 4 *	180 ± 2 *
AHeH	-	-	114 ± 12	161 ± 11 *	192 ± 6 *	166 ± 10 *

Notes: *—Significant differences compared with control animals at *p* < 0.05. Data are expressed as M ± m. M—arithmetic mean as % relative to the control group (100%); m—standard error of the mean as % relative to the arithmetic mean. AHBH acute hypobaric hypoxia; AHwHc acute hypoxia with hypercapnia; AHeH—acute hemic hypoxia; AHtH—acute histotoxic hypoxia.

**Table 2:** Clinical efficacy of using Acyzol in the treatment of pneumonia resulted from of acute poisoning with neurotropic agents.

Indicant	Conventional Treatment	Acyzol
Duration of pneumonia, days	14.1 ± 1.1	13.2 ± 0.1
Lethality of pneumonia, %	43.3	7.7 *

Notes: *—Significant differences compared with the group of patients who received conventional treatment, criterion X_2_ using the Yeats amendment, *p* < 0.05.

**Table 3:** Heart rate and electrocardiogram measurements of rats during the treatment of myocardial infarction with Acyzol, % of normal.

Time from Start of the Treatment	Heart Rate, BPM	P Wave, mV	R Wave, mV	S Wave, mV	T Wave, mV	PQ Interval, ms	QT Interval, ms
Control (off treatment)							
1 h	76.31•	63.29•	259.66•	256.96•	177.47•	141.66•	158.43•
7 days	114.41 *	75.32•	169.23 *•	173.41 *•	122.86 *	138.27•	166.22•
Acyzol, 60 mg/kg							
1 h	106.24	87.47	155.66•	86.61	97.43•	84.25•	99.62•
7 days	108.21	92.73	149.28•	95.78•	104.57•	97.74	109.87

Notes: *—significant differences compared to the observation period of 1 h at *p* < 0.05. •—significant differences compared to normal at *p* < 0.05.

**Table 4:** Index score after 1 month from the start of treatment Acyzol (M ± m).

Group	Type of Treatment	Indicant	OHI-S, Scores	SBI, %
Group I	Acyzol, local application (dental paste + oral rinse) *n* = 20	GMiCP	0.4 ± 0.1	7.1 ± 0.3
		GMoCP	0.7 ± 0.1	10.8 ± 0.5
		GSCP	1.3 ± 0.1	12.3 ± 0.9
Group II	Acyzol, oral *n* = 20	GMiCP	0.2 ± 0.1	4.6 ± 0.2
		GMoCP	0.3 ± 0.1	9.0 ± 0.4
		GSCP	0.6 ± 0.2	10.4 ± 0.5
Group III	Acyzol, oral+ local application (dental paste + oral rinse) *n* = 22	GMiCP	0.2 ± 0.1	5.1 ± 0.8
		GMoCP	0.4 ± 0.1	9.4 ± 0.8
		GSCP	0.8 ± 0.1	10.6 ± 0.4

GMiCP—generalized mild chronic periodontitis; GMoCP—generalized moderate chronic periodontitis; GSCP—generalized severe chronic periodontitis; OHI-S—Oral hygiene index score (J.C.Green, J.R.Vermillion, 1964); SBI—sulcus bleeding index (H.R. Muhlamann в мoдификации I. Cowell, 1975).

**Table 5:** Impact of preventive Acyzol administration on catalase activity and malondialdehyde (MDA) concentration in experimental animals chronically intoxicated with lead acetate.

Group	MDA, μL	*P*	Catalase (mcat/L)	*p*
Group I	76.52 ± 1.769	-	455.5 ± 16.7	-
Group II	89.3 ± 1.74 *	≤0.001	320.1 ± 10.16 *	≤0.001
Group III	82.4 ± 1.3	≤0.01	374.7 ± 12.47	≤0.001

Group I—intact animals; Group II—animals with intragastric administration of lead acetate at a dose of 40 mg/kg for 16 days; Group III—animals with intragastric administration of lead acetate at a dose of 40 mg/kg and subcutaneous administration of Acyzol at a dose of 30 mg/kg for 16 days.
